# Near-Infrared Spectroscopic Method for Monitoring Water Content in Epoxy Resins and Fiber-Reinforced Composites

**DOI:** 10.3390/ma11040586

**Published:** 2018-04-11

**Authors:** Andrey E. Krauklis, Abedin I. Gagani, Andreas T. Echtermeyer

**Affiliations:** Department of Mechanical and Industrial Engineering (past: Department of Engineering Design and Materials), Norwegian University of Science and Technology, 7491 Trondheim, Norway; abedin.gagani@ntnu.no (A.I.G.); andreas.echtermeyer@ntnu.no (A.T.E.)

**Keywords:** epoxy, composites, water, NIR, spectroscopy

## Abstract

Monitoring water content and predicting the water-induced drop in strength of fiber-reinforced composites are of great importance for the oil and gas and marine industries. Fourier transform infrared (FTIR) spectroscopic methods are broadly available and often used for process and quality control in industrial applications. A benefit of using such spectroscopic methods over the conventional gravimetric analysis is the possibility to deduce the mass of an absolutely dry material and subsequently the true water content, which is an important indicator of water content-dependent properties. The objective of this study is to develop an efficient and detailed method for estimating the water content in epoxy resins and fiber-reinforced composites. In this study, Fourier transform near-infrared (FT-NIR) spectroscopy was applied to measure the water content of amine-epoxy neat resin. The method was developed and successfully extended to glass fiber-reinforced composite materials. Based on extensive measurements of neat resin and composite samples of varying water content and thickness, regression was performed, and the quantitative absorbance dependence on water content in the material was established. The mass of an absolutely dry resin was identified, and the true water content was obtained. The method was related to the Beer–Lambert law and explained in such terms. A detailed spectroscopic method for measuring water content in resins and fiber-reinforced composites was developed and described.

## 1. Introduction

Epoxy resins are widely used in composite materials (e.g., fiber-reinforced laminates), adhesives and surface coatings due to their relatively high strength, stiffness, low volatility and shrinkage during the curing process, as well as good adhesive properties. However, it is known that highly crosslinked amine-cured epoxy resins are hydrophilic, and their properties can significantly deteriorate upon water uptake [1,2,3]. It is well established that water uptake is an important factor in the performance and durability of epoxy-based composites, which undergo plasticization and swelling stresses [4]. 

Composite materials are often exposed to water or humid air environments. This is of special interest for offshore applications, since composites are widely used there due to their relatively light weight and high corrosion resistance, especially when compared to steel. Furthermore, the deep-water applications of composites have to be mentioned such as in, for example, risers and tethers [5]. It has been reported that water environments negatively impact the mechanical properties of such materials [6,7,8,9,10,11,12]. It is therefore of high importance to develop a water content monitoring method for such materials exposed to water environments.

Among the methods used for measuring the water content, such experimental techniques as thermogravimetry, differential scanning calorimetry (DSC), ultraviolet (UV) reflection spectroscopy, attenuated total reflection Fourier transform infrared spectroscopy (ATR-FTIR) and Fourier transform near-infrared spectroscopy (FT-NIR) have been reported [3,13,14,15,16,17,18,19,20]. The most common is a gravimetric method, which provides weight gain information upon water uptake or the weight loss upon drying. A significant drawback of the gravimetric method is the necessity to know the mass of the absolutely dry material, which in some cases requires long drying times and a complicated setup to obtain such data. Therefore, an alternative technique for measuring water content in epoxy resins and fiber-reinforced composites is required. NIR spectroscopy, e.g., FT-NIR, is a promising technique for water content monitoring [21,22,23]. Due to the high transmission of NIR light, its applicability to products with sizes in the mm range is usually possible [21]. 

It is common to report water content without accounting for moisture initially present in the material. In reality, some water is already present in the resin uptaken from the air, which has a certain humidity. The spectroscopic method provides a way to deduce the amount of water present initially in the material. Combining initial water content with that from water uptake experiments, the true water content is obtained. True water content and its determination are described further in the work. It is important to know the true water content when one has to look at strength, stiffness and fatigue property changes due to water content [24,25]. It was found that true water content at initial conditions is already 0.63% for the studied epoxy. These conditions are what would usually be denoted as a dry material. The true water content at saturation is 3.44%. Thus, the initial water content is significant and should not be neglected. The provided method allows determining and quantifying it.

Recent improvements of NIR spectrometers, resulting in a dramatic increase in their performance, e.g., acceleration of measuring time, have further lowered the threshold for using this method for process monitoring [21,22]. NIR has a broad range of practical applications, both in the laboratory and industry, the evaluation of diffusant content being one of those. For instance, NIR spectroscopy has long been used in such fields as medicine, food and polymers [21,26,27,28].

Multiple studies of water content evaluation in polymers have been reported. For example, Camacho et al. [26] and Kuda-Malwathumullage et al. [27] constructed NIR spectroscopic models for polyamides (Nylon 6,6), while Muroga et al. developed an NIR model for evaluating the water content of molded polylactide (PLA) under the effect of crystallization [21]. Studies on the determination of the water content of polymer/filler nanocomposites at variable relative humidity have been reported [29,30]. Use of spectroscopic methods for epoxies has also been reported, including use of FT-NIR to study epoxy network interactions with water molecules [15,17,31,32,33,34]. However, despite the described benefits of NIR spectroscopy, and specifically FT-NIR, and the mentioned relevant studies on epoxy-water interactions, the authors believe that a sufficiently detailed and simple-in-application method needs to be reported and discussed, allowing one to evaluate the water content in epoxy resin and epoxy-based composite samples of varying thickness and water content. Thus, the objective of this study is to establish such a model and provide the details of the method for its application in practice. Previous work has only been done on polymers, but not on fiber-reinforced composites. Such work on composites is seen as novel and important for industry. It has not been reported anywhere else to the best knowledge of the authors. 

## 2. Experimental Section

### 2.1. Materials

Amine-cured epoxy: Amine-cured epoxy resin was prepared using reagents Epikote Resin RIMR 135 (Hexion, International: USA/Europe) and Epikure Curing Agent MGS RIMH 137 (Hexion, International: USA/Europe) in a ratio of 100:30 by weight. The mixture was degassed in a vacuum chamber for 0.5 h in order to remove bubbles. The sample mold was prepared using computer numerical control (CNC) machining. Degassed resin was then molded into rectangular-shaped samples, followed by curing at room temperature for 24 h and post-curing in an air oven (Lehmkuhls Verksteder, Oslo, Norway) at 80 °C for 16 h. After samples were post-cured, the resin was removed from the mold’s grooves and cut into the desired length with a vertical bandsaw. After cutting epoxy samples, the precise desired length was obtained by using sandpaper (FEPA P60, grain size 269 µm) to grind the edges. The prepared mold allows sufficient width control within a tolerance of 5%. In order to get samples to the right thickness and enable sufficient thickness control, a metal holder for grinding was prepared and used. The desired thickness was obtained using PHOENIX 2000 (Jean Wirtz, Dusseldorf, Germany) and SiC grinding discs (Struers, Cleveland, OH, USA; FEPA P500, grain size 30 µm). Exicator grease (Riedel-de Haёn, Seelze, Germany) was used to enable sufficient adhesion of the sample with the holder. The sufficient thickness control, correct length and width were ensured within a 5% tolerance. Dog bone-shaped epoxy resin samples used in tensile tests were prepared in a similar way.

Composite laminates: Glass fiber-reinforced epoxy composite laminates were prepared using the vacuum-assisted resin transfer molding (VARTM) process. The composite laminate plate was turned into cylinders of a diameter of 20 mm. The cylinders were then cut into discs of a thickness slightly above 2 mm. The thickness was then adjusted to 2 mm within a 5% tolerance via grinding with a super fine sandpaper (FEPA P800, grain size 21.8 µm).

DI water: Deionized water (0.5–1.0 MΩ·cm) was used for water uptake measurements, produced via the water purification system Aquatron A4000 (Cole-Parmer, Vernon Hills, IL, USA).

### 2.2. Methods

Conditioning of resin and composite samples in water: Water uptake experiments were conducted using a batch system. A heated DI water (60 °C) bath was used for conditioning the samples. Samples were weighed using the analytical scales AG204 (±0.1 mg; Mettler Toledo, Columbus, OH, USA) in order to obtain the mass of the unconditioned samples and placed into the water bath. Samples were conditioned until equilibrium was achieved. Up to and at the saturation point, the samples were taken out of the water bath, weighed and analyzed with an FTIR spectrophotometer in order to obtain spectra at different water contents.

Drying of conditioned resin and composite samples: The drying of saturated samples was performed in a drying cabinet PK-410 (ESAB, London, UK) at 60 °C in air atmosphere with natural convection and relative humidity of 13 RH%.

Determination of resin fraction in composites: The density of neat epoxy resin ($\rho_{resin}$) and glass fiber ($\rho_{glass}$) was 1.1 g/cm^3^ and 2.54 g/cm^3^, respectively. The density of the composite ($\rho_{comp}$) was determined to be 1.97 g/cm^3^ by measuring the mass and dimensions of a large composite block. The volume and mass fractions of neat resin were calculated using Equations (1) and (2), respectively.

(1) $$V_{f_{resin}}=1-V_{f_{glass}}=1-\frac{\rho_{comp}-\rho_{resin}}{\rho_{glass}-\rho_{resin}}\;$$

(2) $$m_{f_{resin}}=\frac{\rho_{resin}\cdot V_{f_{resin}}}{\rho_{resin}\cdot V_{f_{resin}}+\rho_{glass}\cdot \left(1-V_{f_{resin}}\right)}$$

The volume and mass fraction of resin are $V_{f_{resin}}=0.394$ and $m_{f_{resin}}=0.220$, respectively.

FT-IR: Sample characterization with different water content was performed using Fourier transform infrared spectroscopy (FTIR) in the near-infrared range. Near-infrared spectra were obtained using the Fourier transform spectrophotometer NIRSystems 6500 (Foss, Eden Prairie, MN, USA) operated in transmission mode; an optical fiber probe and spectral analysis software Vision (Foss, Eden Prairie, MN, USA) were used. Spectra were taken in the Vis-NIR wavenumber range of 4000–25,000 cm^−1^ using 32 scans per spectrum with a resolution of 4 cm^−1^. A spectrum of the driest (in this case, dried) sample was subtracted from the spectrum of the sample of interest. Then, the line connecting spectrum points at 5400 and 4900 cm^−1^ was constructed. The slope and the intercept of this line were obtained. Subsequently, baseline correction was performed by subtracting the obtained line from the spectrum of interest.

Tensile tests: To evaluate the effect of water content on the ultimate tensile strength (UTS) of neat epoxy resin, tensile tests were conducted using the servo hydraulic test machine Instron Model 1342 (Instron, International: USA/UK). Dog bone-shaped epoxy resin samples were used in order to determine tensile strength; the rate was set to 1 mm/min of controlled displacement.

## 3. Results and Discussion

### 3.1. Water Uptake, Drying and Conditioning in Air Experiments of Neat Resin

The definition of water content in neat resin is described by Equation (3):

(3) $$W\left(t\right)\equiv \frac{m\left(t\right)-m_{dried}}{m_{dried}}\cdot 100\%$$

where m(t) is the mass of the wet neat resin; $m_{dried}$ is the mass of the resin after drying.

**Figure 1:**
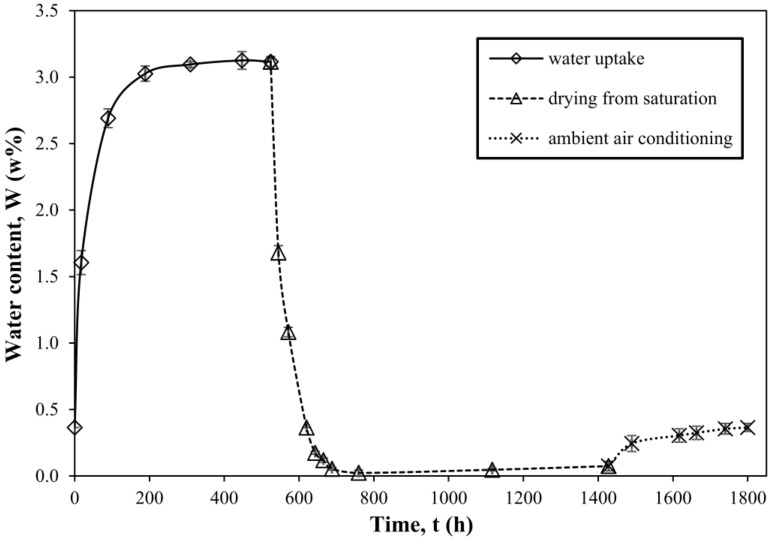
Water uptake, drying and conditioning in air curves of amine-cured epoxy resin.

Four samples of neat resin were used to obtain the water uptake and drying curves, as well as conditioning in air at room temperature back to initial water content; average values with standard deviations are reported. The curves are shown in Figure 1.

### 3.2. Reversible Drop in Ultimate Tensile Strength of Neat Resin with Water Content

**Figure 2:**
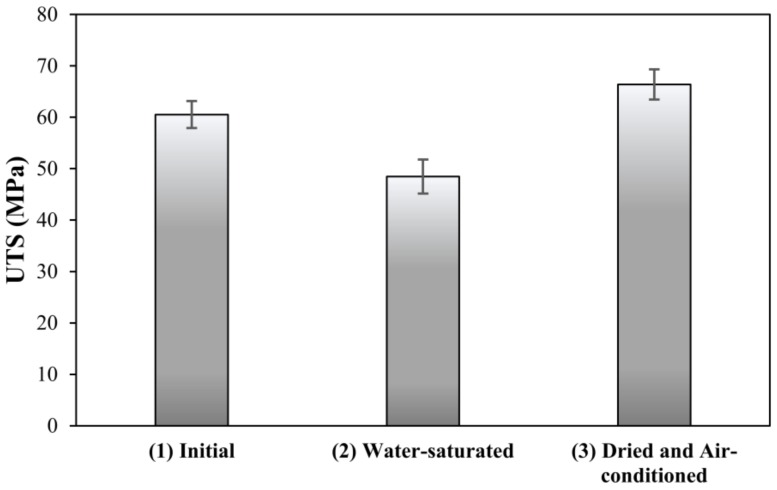
Water-induced drop in ultimate tensile strength (UTS) of amine-cured epoxy resin.

The ultimate tensile strength (UTS) was measured (1) for neat resin at initial conditions (in equilibrium with water vapor in ambient conditions), (2) for water-saturated resin and (3) for dried resin air-conditioned back to initial water content. The ultimate tensile strength (UTS) has decreased down to 80% of the initial dry value (from about 60 down to 48 MPa), as shown in Figure 2. Results indicate that the UTS of dried epoxy is comparable to that of the initial, indicating the reversibility of the drop in UTS. This aspect of reversibility is reported in greater detail elsewhere [24].

### 3.3. The Method for Monitoring Water Content in Neat Resin

**Figure 3:**
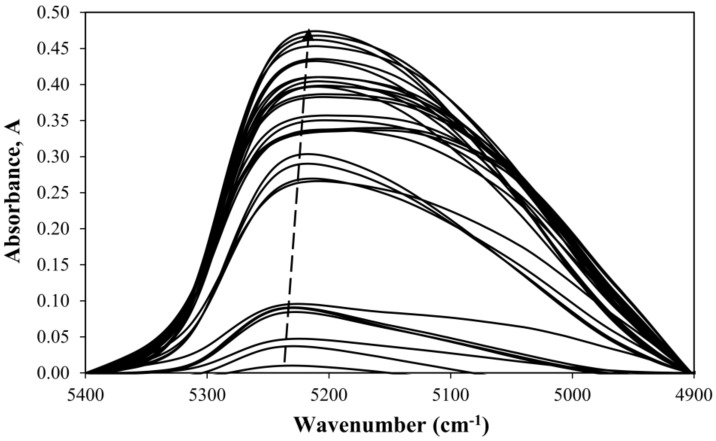
Difference of spectra with respect to the dried epoxy of the water absorbance band in epoxy resin samples of varying water content. The baseline corresponds to the driest sample (in this case, dried).

The spectrum of a dried epoxy sample was obtained and is further denoted as the reference spectrum (the sample with the lowest water content available). The water (diffusant) absorption band’s maximum was identified to be at about 5200 cm^−1^. This band changed depending on the water content, as shown in Figure 3. Based on study by Falk et al. [35], this band corresponds to a combination mode of stretching and bending of the water molecule’s OH group. This observation is also consistent with a recent study by Muroga et al. on spectroscopic evaluation of water content in polylactide (PLA) and with a novel work on water monitoring methods in PMMA by Wiedemair et al. [21,36]. Thus, this absorption band is chosen to monitor the water content of the epoxy resin. The wavenumber of the water absorption band shifts to lower values as the water content increases. True water content and the corresponding wavenumber values at the absorption peak are reported in Table 1.

The absorbance at the maximum of the water absorption band was quantified in the identified range. Absorbance values of the water band are comparable to those reported for studying water content in other polymeric systems [15,21]. The linear regression analysis (linear least square method) was then performed to obtain the equation A=f(W). Based on 40 measurements, for the studied neat resin, the regression equation (Equation (4)) was obtained (R^2^ = 0.9466):

(4) $$A=0.1248W+0.0330=K_{slope}^{0}W+\beta$$

where 0.0330 is the intercept β and is due to the undried water content. Since the slope is the same for the undried water content $W_{undried\;water}$ as for the determined water content W, the relationship can be rewritten in the form of Equation (5).

(5) $$A=K_{slope}^{0}\left(W+W_{undried\;water}\right)$$

When W is equal to zero, absorbance is present because the samples are not fully dried. Thus, the intercept $W_{undried\;water}$ is due to the presence of water that was not removed during the drying process. This allows one to determine the amount of water present in the sample after drying, as shown by Equation (6), and to deduce how much water was in samples at equilibrium at ambient conditions if needed. Furthermore, this allows one to determine the mass of the absolutely dry material.

(6) $$W_{undried\;water}=\frac{\beta }{K_{slope}^{0}}$$

In this case, the undried water content $W_{undried\;water}$ is equal to 0.26%, meaning that even after drying, there is still a significant amount of water present, which is supported by the observable water band in the IR spectra of dried samples. In order to calculate the mass of the absolutely dry neat resin $m_{absolutely\;dry}$ (Equation (8)), Equations (3) and (5) are used.

(7) $$A=K_{slope}^{0}\left(\frac{m\left(t\right)-m_{dried}}{m_{dried}}\cdot 100\%+\frac{W_{undried\;water}m_{dried}}{m_{dried}}\right)$$

(8) $$m_{absolutely\;dry}=\left(1-\frac{W_{undried\;water}}{100}\right)m_{dried}$$

This leads to a definition of the true water content. The true water content shows the amount of water with respect to the absolutely dry material and is defined as in Equation (9).

(9) $$W^{*}\left(t\right)\equiv \frac{m_{water}\left(t\right)}{m_{absolutely\;dry}}\cdot 100\%=\left(\frac{m\left(t\right)-m_{absolutely\;dry}}{m_{absolutely\;dry}}\right)\cdot 100\%$$

The water content monitoring equation then can be written in the form of Equation (10).

(10) $$A=K_{slope}W^{*}$$

The water content is then recalculated to obtain the true water content $W^{*}$ as defined by Equation (9). The linear regression for $A=f\left(W^{*}\right)$ gives a similar slope ($K_{slope}$ is equal to 0.1247) with a zero intercept to the initial result of A=f(W) ($K_{slope}^{0}$ is equal to 0.1248). The determination coefficient is R^2^ = 0.9466, which indicates that this model equation accounts for 89.61% of the variation in absorbance band maximum values in the dataset. The remaining 10.39% of variation not explained by the equation is expected to be due to the sample thickness having a tolerance of 5%, as well as some possible drying in air during the collection of spectra, since spectra are taken in ambient conditions at room temperature in air atmosphere. Such low variation even within a 5% thickness tolerance of samples indicates this method as being precise and efficient for monitoring water content of neat resin. Thus, this method can be used as an indicator of the water concentration-dependent drop of the mechanical properties of the material. 

### 3.4. Extension of the Method to Samples of Varying Thickness

Based on the Beer–Lambert law (Equation (11)), absorbance is dependent on the molar attenuation (absorption) coefficient (ε), concentration (c) and the path length (l).

(11) $$A=\log_{10}\left(\frac{I_{0}}{I}\right)=\varepsilon cl$$

In our case, the concentration term is the true water content ($W^{*}$), and the path length is the thickness of the sample (δ). Thus, the Beer–Lambert law in our case can be expressed as Equation (12).

(12) $$A=\varepsilon^{*}W^{*}\delta$$

Note that in this case, attenuation coefficient $\varepsilon^{*}$ is not the same as a conventional molar attenuation coefficient ε, since the concentration term used is of true water content ($W^{*}$) and not of the molar concentration (c); thus, in order to avoid misunderstanding, in this work, the proportionality coefficient will be denoted as $\varepsilon^{*}$. However, calculation of the molar attenuation coefficient will be shown later in this work, as well. In practice, mass concentrations are easier to imagine and work with. However, conventionally, attenuation coefficients are given in molar units. Thus, a way to convert between mass and molar concentrations is provided.

Since the model is as shown in Equation (10), the thickness effect (path length) is contained in the slope term of the model equation (Equation (9)); thus, the slope term can be divided into the attenuation coefficient and the thickness. The new slope term is the attenuation coefficient and is obtained as shown in Equation (13).

(13) $$\varepsilon^{*}=\frac{K_{slope}}{\delta }$$

**Figure 4:**
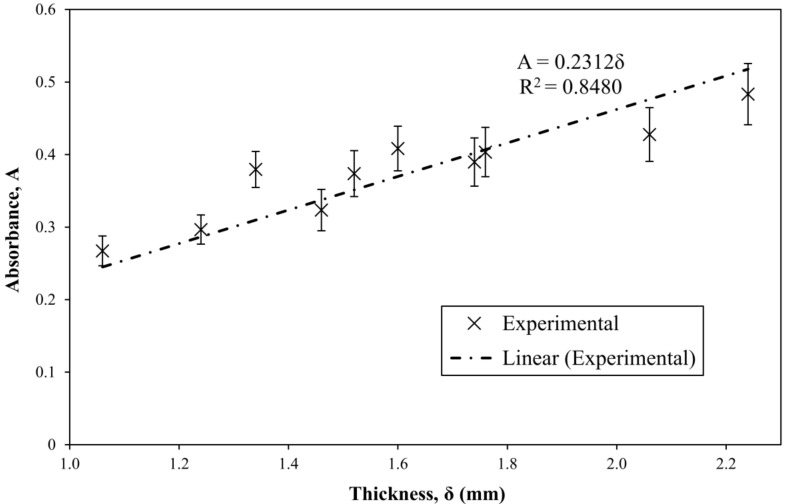
Linear dependence of absorbance on the sample thickness of water-saturated neat resin.

In the case of resin, the water content monitoring equation (Equation (9)) then becomes Equation (12). The model in Equation (12) is validated by using water-saturated resin samples of varying thickness, e.g., by setting term $W^{*}$ to a constant value, thus proving the linear dependence of absorbance on sample thickness (path length), as shown in Figure 4. The determination coefficient is R^2^ = 0.8480.

Based on the linear dependence of absorbance on sample thickness, for saturated samples $\varepsilon^{*}W_{max}^{*}$ equals 0.2312, and the attenuation coefficient is then 0.0672 ± 0.0034${\%}^{-1}\cdot {\mathrm{mm}}^{-1}$ (R^2^ = 0.8480). Using model equation (Equation (10)), it is known that $\varepsilon^{*}\delta_{2\;mm}$ equals 0.1247. From this, taking into account a 5% thickness tolerance, the attenuation coefficient is 0.0624 ± 0.0031${\%}^{-1}\cdot {\mathrm{mm}}^{-1}$. The values are within the standard deviation. Since the attenuation coefficient $\varepsilon^{*}$ in our case has units of ${\%}^{-1}\cdot {\mathrm{mm}}^{-1}$, in order to obtain the molar attenuation coefficient ε, calculation of water molar concentration is required. Using the definition of true water content (Equation (9)), the relationship between the molar concentration of the diffusant (water) and the true water content can be written as Equation (14).

(14) $$c\left(t\right)\left[\frac{mol}{L}\right]=\frac{\left[\frac{\frac{W^{*}\left(t\right)\;\left[\%\right]}{100}\cdot m_{absolutely\;dry}\left[g\right]}{M_{water}\;\left[\frac{g}{\mathrm{mol}}\right]}\right]}{a\cdot b\cdot \delta \;\left[{\mathrm{mm}}^{3}\right]\cdot 10^{-6}\;\left[\frac{L}{{\mathrm{mm}}^{3}}\right]}$$

where *a* and *b* are length and width of the sample in mm, respectively.

Since thickness is the path length (l = δ) and $\varepsilon cl=\varepsilon^{*}W^{*}\delta$ (Equations (11) and (12)), the molar attenuation coefficient can then be obtained from Equation (15).

(15) $$\varepsilon =\frac{\varepsilon^{*}W^{*}}{c}$$

Since $\varepsilon^{*}W^{*}$ is found to be equal to 0.2312 mm^−1^ for the resin of interest at full saturation ($W_{max}^{*}=3.44\%$) for a sample of 2 mm in thickness, using Equation (14), the molar concentration is 2.29 ± 0.12 M, and using Equation (15), the molar attenuation coefficient ε is equal to 1.01 ± 0.06 $\frac{L}{\mathrm{mol}\cdot \mathrm{cm}}$. The low value of the molar attenuation coefficient is explained by the fact that resin media has a relatively high light attenuation itself. Note that molar attenuation coefficient for water in epoxy media ε is obtained using the difference spectra with respect to the spectrum of the dried neat resin.

The authors have assessed the method in the thickness range from 1.06–2.24 mm. The authors expect that the method is applicable also to thinner samples. Extrapolation to samples thicker than 2.24 mm is not completely certain.

### 3.5. Extension of the Method to Composite Systems

In order to extend the method of monitoring water content to fiber-reinforced composites, the mass fraction of resin has to be known. For the studied glass-fiber reinforced composites, the resin mass fraction for samples at initial conditions ($m_{f_{resin_{initial}}}$) was determined as described earlier and is 0.2198. The resin mass fraction is used in order to deduce diffusant uptake by resin from the composite spectra. The proposed equation for monitoring the true water content due to exposure of composites to water media is represented by Equation (16).

(16) $$A=\varepsilon^{*}W_{resin}^{*}\delta =K_{slope}\;W_{resin}^{*}$$

The definition of the water content in the composite material is described by Equation (17).

(17) $$W_{comp}\equiv \frac{m\left(t\right)-m_{comp_{dried}}}{m_{comp_{dried}}}\cdot 100\%$$

The true water content of composites is defined by Equation (18).

(18) $$W_{comp}^{*}\equiv \frac{m_{comp}\left(t\right)-m_{comp_{absolutely\;dry}}}{m_{comp_{absolutely\;dry}}}\cdot 100\%$$

An assumption is required that fiber is always dry (not absorbing water) and neglecting the influence of the sizing, since the mass of sizing is negligible compared to resin mass. This is, however, not always fully true and is a convenient assumption. The mass of the composite is then as shown in Equation (19).

(19) $$m_{comp_{dried}}=m_{resin_{dried}}+m_{fiber}$$

**Figure 5:**
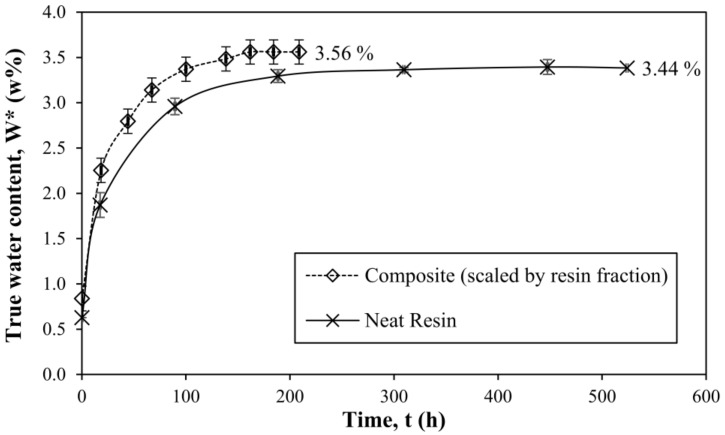
True water content curves of neat resin and glass fiber-reinforced composite (scaled by the resin mass fraction).

In order to prove that the assumption is valid in this case, that the increase in fiber or sizing mass is negligible due to water uptake, the water uptake using prepared composite discs was performed and scaled by the factor of the resin mass fraction of the composite. The comparison of the neat resin and the scaled composite water uptake graphs is represented in Figure 5. 

The shape of the kinetic curve is sample geometry-dependent and is slightly different in this case, as seen in Figure 5. However, the equilibrium water uptake value is a material property and can be used for the comparison. As seen in Figure 5, the equilibrium true water content value for a composite (scaled by resin mass fraction) is only slightly higher than that for the neat resin, resulting in a difference of 0.12% in true water content between average values. The difference can be explained due to the sizing also absorbing water. The authors believe that an increase of the true water content (at equilibrium) for the composite compared to the epoxy is increased due to the interactions of epoxy/fibers via the formation of the interphase. It is likely that what is measured is a sum of water content in both resin and the interphase. It has been reported that the interphase might be responsible for increased water uptake [37]. 

Thus, the assumption that only resin uptakes water is not completely true, but considering the relatively low difference, this is a reasonable approximation. Another aspect that might be causing the difference in the equilibrium water uptake value could also be the presence of voids in the composite, which is usually greater than in the neat resin due to the specifics of the impregnation process.

Use of such and approximation should be assessed in the case of other fiber-resin systems, since sizing can have a varying influence on the water uptake in different systems. For instance, in the case of carbon fiber-vinylester composites, it has been reported that the sizing affects the equilibrium water uptake of the composite significantly [37]. Thus, it has to be noted that the extension of the method might have limitations for certain composite systems.

Since the mass of water comes into the resin mass term, the mass fraction of resin is dependent on the water content and is defined as in Equation (20).

(20) $$m_{f_{resin}}\left(t\right)\equiv \frac{m_{resin}\left(t\right)}{m_{resin}\left(t\right)+m_{fiber}}=\frac{m_{resin_{absolutely\;dry}}+m_{water}\left(t\right)}{m_{resin_{absolutely\;dry}}+m_{water}\left(t\right)+m_{fiber}}$$

In order to obtain the absolutely dry mass of the composite material, the set of Equations (21)–(23) is used.

(21) $$m_{f_{resin_{dried}}}=\frac{m_{comp_{dried}}-\left(1-m_{f_{resin_{initial}}}\right)\cdot m_{comp_{initial}}}{m_{comp_{dried}}}$$

(22) $$m_{resin_{absolutely\;dry}}=m_{f_{resin_{dried}}}\cdot m_{comp_{dried}}\cdot \left(1-\frac{W_{undried\;water}}{100}\right)$$

(23) $$m_{comp_{absolutely\;dry}}=m_{resin_{absolutely\;dry}}+\left(1-m_{f_{resin_{initial}}}\right)\cdot m_{comp_{initial}}$$

The true water content of the resin component in the composite can be calculated using Equation (24).

(24) $$W_{resin}^{*}=\frac{m_{comp}\left(t\right)-\left(1-m_{f_{resin_{initial}}}\right)\cdot m_{comp_{initial}}-m_{resin_{absolutely\;dry}}}{m_{resin_{absolutely\;dry}}}\cdot 100\%$$

Calculated $W_{resin}^{*}$ is then to be used for predicting the water content, using Equation (25), analogous to the monitoring equation for neat resin (Equation (10)).

(25) $$A=K_{slope}\;W_{resin}^{*}$$

In order to use the composite spectra for predicting $W_{resin}^{*}$, a factor for scaling from composites down to resin is required. Since composite materials have components of different absorbance, a normalization procedure is required. The composite absorbs light much more than the neat resin. There is a necessity to correct for the summary absorbance of the components via the baseline shift. In order to do so conveniently, two parameters need to be determined for the developed absorbance band maximum-based model: the maximum absorbance of the neat resin ($A_{peak_{resin}}$) and of the composite ($A_{peak_{comp}}$) at water saturation (at water uptake equilibrium values). These values are obtained using the difference spectra for respective fully-saturated materials as described earlier. In this case, the obtained values are $A_{peak_{resin}}$ = 0.47 and $A_{peak_{comp}}$ = 0.044. Note that due to the higher absorbance of the composite material, the absorbance of the water band deduced from the difference spectra is one order of magnitude lower than for the neat resin. In order to extend the model from neat resin to composites, we introduce the scaling factor, which is denoted as Peak Factor and defined as shown in Equation (26). In our case, it is equal to 0.094.

(26) $$Peak\;Factor\equiv \frac{A_{peak_{comp}}}{A_{peak_{resin}}}$$

The final water content monitoring equation for the composite system can then be written in the form of Equation (27) if the effect of thickness is known and in the form of Equation (28) if the effect of thickness is not known.

(27) $$A_{peak_{comp}}=\varepsilon^{*}\cdot Peak\;Factor\cdot \delta \cdot W_{resin}^{*}$$

(28) $$A_{peak_{comp}}=K_{slope}\cdot Peak\;Factor\cdot W_{resin}^{*}$$

For the particular composite system of interest (glass fiber-reinforced amine-cured epoxy), the water monitoring equation is represented by Equation (29).

(29) $$A_{peak_{comp}}=0.012\cdot W_{resin}^{*}$$

**Figure 6:**
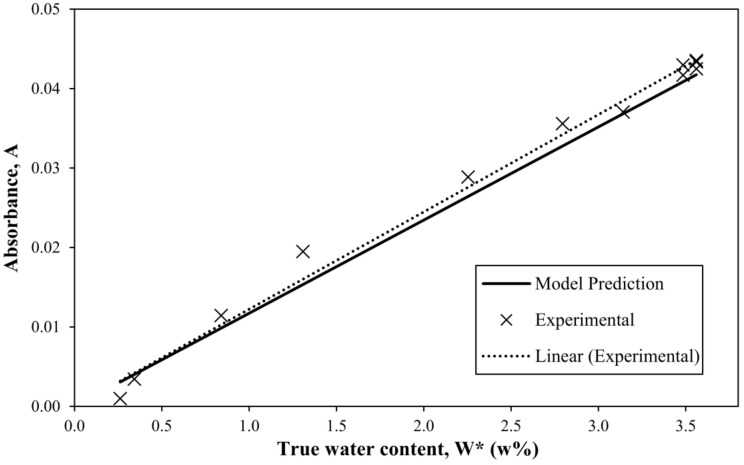
Validation of the water monitoring model extended to composites.

The model was validated by the experimental data using composite discs with a varying water content. The experimental data and predicted values (Equation (29)) are shown in Figure 6.

The fit between the final model and experimental data for composites resulted in a determination coefficient R^2^ of 0.9329, meaning that the developed model accounts for 87.03% of the variation in the absorbance band’s maximum values in the dataset. The best linear regression fit (R^2^ = 0.9908) has only a slightly higher slope than the model, as shown in Figure 6. 

### 3.6. Final Remarks on the Results

The developed method is potentially useful also for other polymers and composite systems with different fiber or resin fractions, as well as for diffusant media other than water. The developed method is based on the physics behind the Beer–Lambert law and can be used for samples of varying thickness. It should be noted, however, that the method has its limitations. If the sample is too thick or non-transparent in the studied radiation wavelength range, the method is not applicable, e.g., monitoring of carbon fiber-composite materials is expected to be limited.

The method can be used as an indicator of water-induced property changes in polymers and composites. While the drop in ultimate tensile strength (UTS) of the neat resin is reversible for the studied amine-cured epoxy resin, some types of fibers, such as glass fibers, are susceptible to irreversible degradation. Thus, in composites, the decline of UTS is not only water-concentration dependent, but also time-dependent. In cases when the material stays in the water environment during its working conditions, the driving force is always directed towards the drier interior of the material. In such cases, spectroscopic water content monitoring can provide valuable information about the interaction time of composite constituents (matrix, fibers, sizing) with the diffusant.

In practice, when the water content distribution in a composite structure is of interest, use of a transmittance FTIR is very limited. In order to become applicable for such cases, the following scenario is proposed. A replica of the structure parts, alongside the actual structure, should be immersed in water media at the same conditions. These replica parts then can be cut into samples of the thickness applicable for the method and analyzed. While destructive and requiring additional expenses, the method can be a useful indicator of long-term water content and distribution in the composite structures, where such destructive tests might be required only once in tens of years.

Other spectroscopic methods such as Raman, while not as sensitive to water, and reflectance FTIR, while mostly providing information about the surface of the material, might also be considered in developing water monitoring methods, especially in cases when composites are non-transparent to the IR radiation.

## 4. Conclusions

In this study, a detailed method for estimating and monitoring water content in epoxy resins and fiber-reinforced composites was developed using the maxima of the absorbance band at about 5200 cm^−1^ in the NIR combination mode region correlated with the true water content. The method provides a benefit over the conventional gravimetric analysis providing the possibility to deduce the mass of an absolutely dry material and subsequently the true water content, which is an important indicator of water content-dependent properties. Based on extensive measurements of neat resin and composite samples of varying water content and thickness, regression was performed, and the quantitative absorbance dependence on water content in the materials was successfully established. The model equations for monitoring water content in epoxy resin and composite material samples were obtained and experimentally validated. The model was related to the Beer–Lambert law and explained in such terms. The details of the method were reported, allowing the use of the method in practical applications.

## Figures and Tables

**Table 1:** True water content and the corresponding wavenumber values at the absorption peak.

True Water Content, W* (%)	Wavenumber (cm^−1^)
0.26	5230
0.61	5225
1.68	5219
1.88	5214
2.84	5214
2.94	5208
3.34	5208
